# Supplementary material for: Delivery of A Chemically Modified Noncoding RNA Domain Improves Dystrophic Myotube Function
Source: Adv Sci (Weinh). 2025 Feb 17;12(20):2410908. doi: 10.1002/advs.202410908 (PMC12120708; doi:10.1002/advs.202410908)
Supplement: Supplementary file 1 — Supporting Information [file ADVS-12-2410908-s001.docx]

***Advanced Science***

Supplementary Information for

**Delivery of a chemically modified noncoding RNA domain improves dystrophic myotube function**

Niasse-Sy *et al.* 2025

Corresponding author

E-mail: [mwohl@mit.edu](mailto:mwohl@mit.edu)

**This file includes:**

Supplementary Figures S1 to S2

Supplementary Tables S1 to S4

**Supplementary Figures**

**Figure S1.** Human CYTOR exon 1 and exon 2. (**A**) CYTOR RNA isoform expressed across 54 human tissues in the Genotype-Tissue Expression (GTEx) portal (accessible on: https://gtexportal.org/home). (**B-C**) SHAPE directed RNA secondary structure prediction of full length CYTOR (**B**) in vitro and (**C**) in cellulo from K562 cells. (**D**) Box plots showing SHAPE reactivity distributions for CYTOR exon 1 and exon 2.

**
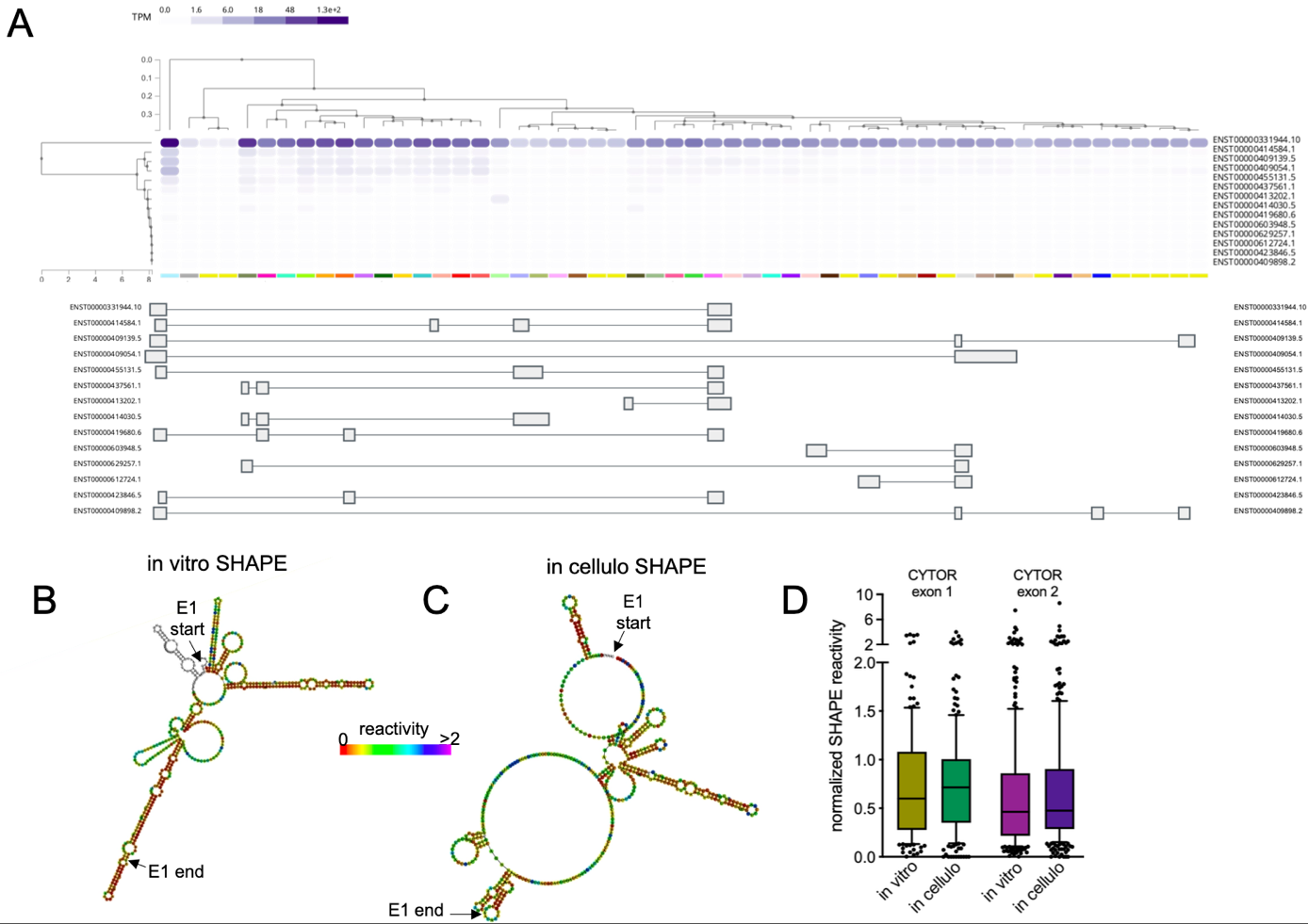
**

**Figure S2.** (**A**) Nucleic acid concentration over 24h of CYTOR plasmid or CYTOR exon 2 RNA in standard cell culture medium at 37C. Concentrations were measured with nanodrop. N=4. (**B**) Normalized abundance of *TNFα* and *IL-6* in skeletal muscle myotubes from Duchenne muscular dystrophy patients treated with chemically modified CYTOR exon 2 RNA or scramble control RNA. N=8. (**C**) Cell viability and apoptosis in dystrophic myotubes and healthy myotubes treated with scramble control RNA, or chemically optimized CYTOR exon 2 RNA. N=8. *P < 0.05, **P < 0.01, ***P<0.001.


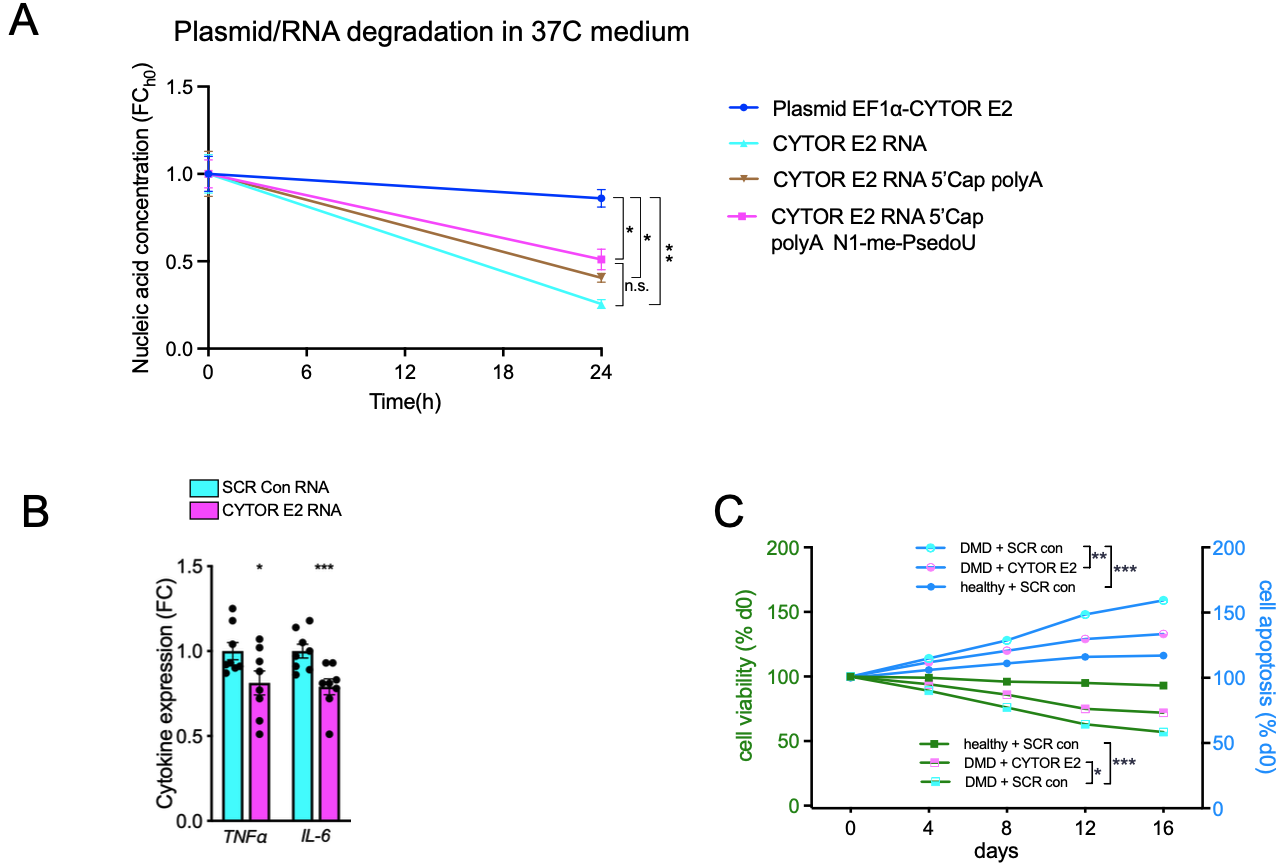


**Supplementary Tables**

**Table S1.** Human CYTOR exon sequences

>ENST00000331944.10 CYTOR-201 FULL LENGTH cdna:lncRNA

GTTCCAATGAGAATGAAGGCTGAGGTGTGCGCCTTTTTTTTTTTTTCCTTCTTAGTCGTGTGTACATCATTGGGAATGGAGGGAAATAAATGACTGGATGGTCGCTGCTTTTTAAGTTTCAAATTGACATTCCAGACAAGCGGTGCCTGAGCCCGTGCCTGTCTTCAGATCTTCACAGCACAGTTCCTGGGAAGGTGGAGCCACCAGCCTCTCCTTGAATAACTGGGAGATGAAACAGGAAGCTCTATGACACACTTGATCGAATATGACAGACACCGAAAATCACGACTCAGCCCCCTCCAGCACCTCTACCTGTTGCCCGCCGATCACAGCCGGAATGCAGCTGAAAGATTCCCTGGGGCCTGGTTCCAACCGCCCACTGTGGACTCTGAGGCCTCTGCATTTGCGGGTGGTCTGCCTGTGATATTTTGGTCATGGGCTGGTCTGGTCGGTTTCCCATTTGTCTGGCCAGTCTCTATGTGTCTTAATCCCTTGTCCTTCATTAAAAGCAAAACTAAAGAAAACAGAA

>ENST00000331944.10 CYTOR-201 EXON1 cdna:lncRNA

GTTCCAATGAGAATGAAGGCTGAGGTGTGCGCCTTTTTTTTTTTTTCCTTCTTAGTCGTGTGTACATCATTGGGAATGGAGGGAAATAAATGACTGGATGGTCGCTGCTTTTTAAGTTTCAAATTGACATTCCAGACAAGCGGTGCCTGAGCCCGTGCCTGTCTTCAGATCTTCACAGCACAGTTCCTGGGAAGGTGGAGCCACCAGCCTCTCCTTG

>ENST00000331944.10 CYTOR-201 EXON2 cdna:lncRNA

AATAACTGGGAGATGAAACAGGAAGCTCTATGACACACTTGATCGAATATGACAGACACCGAAAATCACGACTCAGCCCCCTCCAGCACCTCTACCTGTTGCCCGCCGATCACAGCCGGAATGCAGCTGAAAGATTCCCTGGGGCCTGGTTCCAACCGCCCACTGTGGACTCTGAGGCCTCTGCATTTGCGGGTGGTCTGCCTGTGATATTTTGGTCATGGGCTGGTCTGGTCGGTTTCCCATTTGTCTGGCCAGTCTCTATGTGTCTTAATCCCTTGTCCTTCATTAAAAGCAAAACTAAAGAAAACAGAA

**Table S2.** List of mouse and human qPCR primers.

| **Gene symbol (mouse)** | **Forward** | **Reverse** |
| --- | --- | --- |
| *Myod1* | AGCACTACAGTGGCGACTC | GTGGAGATGCGCTCCACT |
| *Pax7* | TCTCCAAGATTCTGTGCCGAT | CGGGGTTCTCTCTCTTATACTCC |
| *Myf5* | TGAGGGAACAGGTGGAGAAC | TGGAGAGAGGGAAGCTGTGT |
| *Myf6* | AGATCGTCGGAAAGCAGC | CCTGGAATGATCCGAAACAC |
| *Myog* | TTGCTCAGCTCCCTCAACCAGGA | TGCAGATTGTGGGCGTCTGTAGG |
| *Myh4* | ACAAGCTGCGGGTGAAGAGC | CAGGACAGTGACAAAGAACG |
| *Myh1* | CCAAGTGCAGGAAAGTGACC | AGGAAGAGACTGACGAGCTC |
| *Myh2* | CAGAGGCAAGTAGTGGTGGA | CAAATTCTCTCTGAACAGGGCA |
| *Myh4* | ACACAGAGTCAGGCGAGTTT | CAGTGCGTTCTTGGCCTT |
| *Myh7* | GTGGCTCCGAGAAAGGAAG | GAGCCTTGGATTCTCAAACG |
| *Myl1* | AGAGGTAGAAGCGTTGCTGG | GGCCAGTCTTCCCCAACATT |
| *MYOD* | TCTCCTTGGTGTAGGCTCAG | CCTGACCTTGAACGTGAATC |
| *MYOG* | TTGCTCAGCTCCCTCAACCAGGA | TGCAGATTGTGGGCGTCTGTAGG |
| *MYF5* | TGTGGCTCTCTCTCCGTATG | AATACAGACATGCAGGCTTCAC |
| *MYF6* | GTGGAGGAAGTGGTGGAGAA | ACTTTTCGGTCTGGGTTCCT |
| *MYH1* | TGTCTCCAAAGCCAAGGGAAA | CCCTCGAGAGCTGTGAAACT |
| *MYH2* | GTCCTGCTTTAAAAAGCTCCAAGA | TCAAAGGGCCTATTCTGGGC |
| *MYH7* | TTGGCCCCTTTCCTCATCTGT | ATCAGGCACGAAGACATCCTT |
| *MYH4* | GCTGAAGAGGCTGAGGAACA | CCCGACTCTTCACTCTCAGC |
| *MYL1* | AACCACCACTCCTCTTCCAA | AGGGTGGGTTAAAAAGAGAAGGA |

**Table S3.** List of plasmids.

| **Plasmid** | **Reference** |
| --- | --- |
| pLV-EF1a-GFP | this paper |
| pLV-EF1a-CYTOR | this paper |
| pLV-EF1a-CYTOR-E1 | this paper |
| pLV-EF1a-CYTOR-E2 | this paper |
| psPAX2 | Addgene # 12260 |
| pMD2G | Addgene # 12259 |

**Table S4.** List of antibodies.

| **Antibody** | **Supplier** | **Reference #** |
| --- | --- | --- |
| Anti-Myosin light chain 1 | Thermofisher | # PA5-29635 |
| Donkey anti-Rabbit IgG secondary antibody | ThermoFisher Scientific | # A-21206 |
